# Co-Cultivation of Two *Bacillus* Strains for Improved Cell Growth and Enzyme Production to Enhance the Degradation of Aflatoxin B_1_

**DOI:** 10.3390/toxins13070435

**Published:** 2021-06-22

**Authors:** Le Wang, Wei Huang, Yu Sha, Haicheng Yin, Ying Liang, Xin Wang, Yan Shen, Xingquan Wu, Dapeng Wu, Jinshui Wang

**Affiliations:** 1 College of Biological Engineering, National Engineering Laboratory for Wheat & Corn Further Processing, Henan University of Technology, Zhengzhou 450001, China; lewang@mail.haut.edu.cn (L.W.); weihuang@mail.haut.edu.cn (W.H.); yusha@mail.haut.edu.cn (Y.S.); Yingliang@mail.haut.edu.cn (Y.L.); Xinwang@mail.haut.edu.cn (X.W.); Yanshen@mail.haut.edu.cn (Y.S.); Xingquanwu@mail.haut.edu.cn (X.W.); 2 School of Environment, Henan Normal University, Xinxiang 453001, China; dapengwu@mail.htu.edu.cn

**Keywords:** degradation of aflatoxin B_1_, two *Bacillus* strains, co-cultivation, detoxifying enzymes production, LC-MS

## Abstract

*Bacillus* sp. H16v8 and *Bacillus* sp. HGD9229 were identified as Aflatoxin B_1_ (AFB_1_) degrader in nutrient broth after a 12 h incubation at 37 °C. The degradation efficiency of the two-strain supernatant on 100 μg/L AFB_1_ was higher than the bacterial cells and cell lysate. Moreover, degradations of AFB_1_ were strongly affected by the metal ions in which Cu^2+^ stimulated the degradation and Zn^2+^ inhibited the degradation. The extracellular detoxifying enzymes produced by co-cultivation of two strains were isolated and purified by ultrafiltration. The molecular weight range of the detoxifying enzymes was 20–25 kDa by SDS-PAGE. The co-culture of two strains improved the total cell growth with the enhancement of the total protein content and detoxifying enzyme production. The degradation efficiency of the supernatant from mixed cultures increased by 87.7% and 55.3% compared to *Bacillus* sp. H16v8 and HGD9229, individually. Moreover, after the degradation of AFB_1_, the four products of the lower toxicity were identified by LC-Triple TOF-MS with the two proposed hypothetical degradation pathways.

## 1. Introduction

The exposition of Aflatoxin B_1_ (AFB_1_) from contaminated food and feed is a serious threat to human and animal health because of its carcinogenicity, hepatotoxicity and immunosuppression and causes economic losses in agriculture on a global basis [1,2,3,4]. Many physical and chemical measures have been taken to remove or eliminate AFB_1_ [5]. However, the required effectiveness, safety, and nutritional retention have not been achieved [6]. Biological methods have attracted widespread attention because of their many advantages, for example, small losses in product quality, safety, economic and environmental protection, etc. [7,8]. Bioremediation, as a friendly environmental remediation method, can convert toxic substances into nontoxic substances or small-molecule chemicals for assimilation [9]. Microbial degradation has become an effective alternative to aflatoxin reduction because it can degrade and remove toxic substances under mild conditions to avoid the loss of nutritional value of food [10].

Studies have shown that bacteria and fungi can reduce aflatoxin by biodegradation [11,12]. It was found that the acellular extracts and liquid cultures of all four bacterial isolates of *Rhodococcus erythropolis* and *Mycobacterium fluoranthenivorans* could effectively degrade AFB_1_ [13]. In addition, Farzaneh found that the supernatant of *B. subtilis* UTBSP1 had a higher degradation rate of AFB_1_ than cells or cell lysate [14]. AFB_1_ was metabolized into degradation products by the extracellular part of *Rhodococcus erythropolis*, and its chemical properties were different than those of AFB_1_ [15]. Samuel reported that AFB_1_ was biodegraded and subsequently converted into to AFD_1_, AFD_2_, and AFD_3_ by *Pseudomonas putida* [16]. *Armillariella tabescens* could effectively degrade aflatoxin B_1_ by detoxification enzymes [17]. MnP could be purified from *Phanerochaete sordida* YK-624, which could be used to catalyze the conversion of biphenyl ring of AFB_1_ to AFB-8,9-dihydrodiol [18]. The mechanism of AFB_1_ degrading by *Tetragenococcus halophilus* CGMCC 3792 may be first absorbed and then degraded by enzyme [19]. Several aflatoxin B_1_ degrading enzymes, such as laccases, peroxidases, oxidases, and reductases, have been studied and identified [20,21,22,23]. In the disparate supernatants shown [14], there are few studies that have identified the enzymes involved.

Mixed cultures composed of different well-designed microorganisms had received widespread attention because individual microorganisms in mixed cultures could be easily modified, and the interaction between microorganisms was used in the functional integration of effective production of biological products [24]. Many studies have centered on the isolation of AFB_1_-degrading microorganisms. Nevertheless, most of the selected microorganisms have a weak ability to degrade AFB_1_. Bioprocesses using finely designed mixed-microbial culture combinations have a higher potential to produce useful biological materials than conventional pure culture processes.

In this study, we screened two kinds of *Bacillus* strains, from which were constructed the mixed culture combination for improving the degradation ability of AFB_1_. The factors affecting the degradation efficiency of AFB_1_ were discussed. Moreover, the mechanism of degrading AFB_1_ by the mixed bacterial cultures was explored. Furthermore, the method of mixed culture provided a novel way to enhance AFB_1_ detoxification. It could be considered an environmentally friendly process for AFB_1_ degradation in food and feed industry.

## 2. Results and Discussion

### 2.1. Screening of Microbial Strains for AFB_1_ Degradation

Both of two strains were isolated from soil samples by the selective culture method. The typical characteristics of *Bacillus* sp. were observed under a biological microscope. They were the rod-shaped gram-positive bacteria, which could form opaque milky white colonies on Luria Bertani plates.

In light of 16S rRNA gene sequences analysis, the relationship between the isolated strain H16v8 and *Bacillus velezensis* was the closest, with a similarity of 99%. *Bacillus* sp. HGD9229 was 99% similar to *Bacillus megaterium* (Figure S1). The partial 16S rRNA gene sequences of two bacteria were submitted to the GenBank database. The accession numbers are MF445154 and MF457663. Moreover, the plate confrontation experiments of the two *Bacillus* were carried out to determine the characteristics of them.

In Figure S2a, no mutual growth inhibition was observed between *Bacillus* sp. H16v8 and *Bacillus* sp. HGD9229 after the incubation on nutrient broth (NB) plate. The results showed that the two *Bacillus* species could be co-cultured in the same medium. As shown in Figure S2b, under the same culture conditions, the cell dry weight of the cells obtained by the mixed culture was higher than the pure culture of each bacterium, indicating that co-culture was more conducive to cell growth. It has been reported that *Bacillus* sp. JCM 9141 and *Bacillus* sp. JCM 9156 could grow together during co-culture, resulting in increased cell growth and enhanced enzyme production capacity, which was related to the competition between the two *Bacillus* species for carbon sources [25].

### 2.2. Degradation of AFB_1_ by Mixed Bacterial Cultures

**Figure 1:**
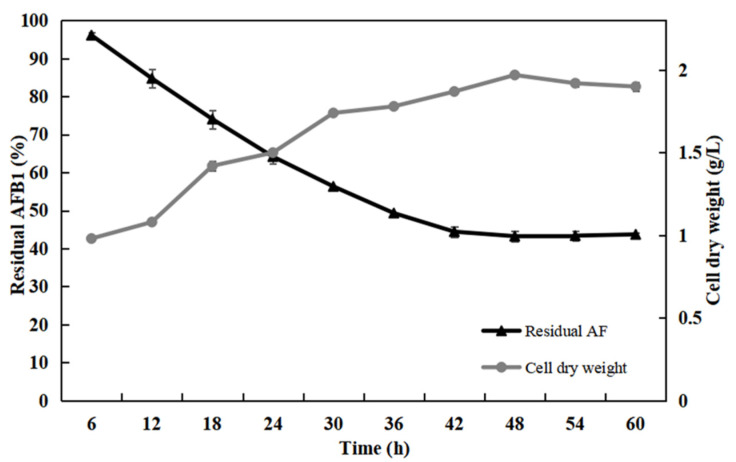
Relationship between degradation rate of Aflatoxin B_1_ (AFB_1_) and growth of *Bacillus* sp. H16v8 and *Bacillus* 423 sp. HGD9229. The AFB_1_ degradation rate and cell dry weight of mixed bacterial cultures were tested at different culture times, including 6, 12, 18, 24, 30, 36, 42, 48, 54, and 60 h, which was cultured in nutrient broth (NB) 37 °C, 180 rpm. All of them incubated 12 h at 37 °C in the dark without shaking. The initial concentration of AFB_1_ was 100 μg/L. The values were the average of three replicates and their standard errors. The mean values were significantly different according to the Duncan’s multirange test (*p* < 0.05).

From the Figure 1, with the increase in co-culture time of *Bacillus* sp. H16v8 and *Bacillus* sp. HGD9229, the bacterial concentration gradually increased, with the decreased residue of AFB_1_. After 48 h of co-culture, the degradation ability of AFB_1_ reached the highest level. At this time, AFB_1_ was degraded by co-culture supernatant, and the maximum degradation rate was 56.7%. An increase in bacterial concentration may enable bacteria to produce more effective detoxifying substances, thus more effectively degrading AFB_1_ [26]. Therefore, the optimal co-culture time of the two *Bacillus* species was 48 h.

### 2.3. Effect of AFB_1_ Detoxification with Mixed Bacterial Cultures

The degradation of AFB_1_ by the mixed bacterial cultures in vitro was displayed in Figure S3. TLC results showed that, compared to the control, the treated samples disappeared the fluorescence of AFB_1_ after the treatment with mixed bacterial cultures. Meanwhile, each pure culture of *Bacillus* sp. H16v8 and *Bacillus* sp. HGD9229 displayed no significant loss of the fluorescence (Figure S3a). It was agreement with the literature [13]. In addition, the degradation rate of AFB_1_ by the mixed bacterial cultures was significantly higher than that of from *Bacillus* sp. H16v8 and *Bacillus* sp. HGD9229, separately (Figure S3b).

### 2.4. Degradation of AFB_1_ by Culture Supernatant, Bacterial Cells, and Cell Lysate

**Figure 2:**
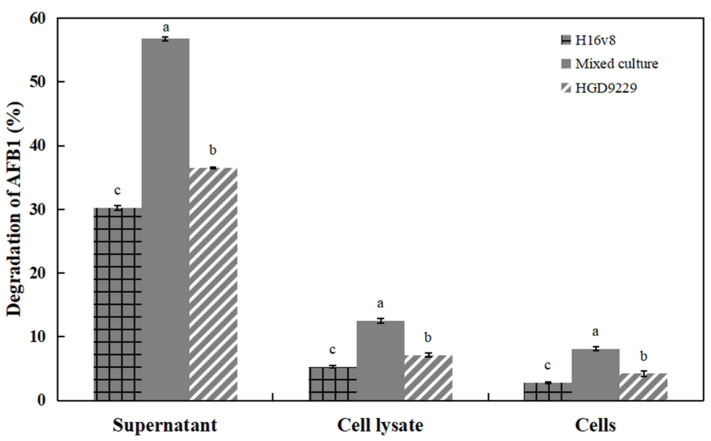
Degradation of AFB_1_ by the culture supernatant, cells and cell lysate of pure and mixed bacterial cultures at 37 °C, 100 μg/L initial AFB_1_ concentration after 12 h incubation. The values were the average of three replicates and their standard errors. The mean values with the different letters were significantly different according to the Duncan’s multirange test (*p* < 0.05).

In Figure 2, the culture supernatant of mixed bacterial cultures had a higher degradation of AFB_1_ (56.7%) after 12 h incubation. Meanwhile, only 12.5% and 8.1% of the degradation rates were obtained when the AFB_1_ was treated with the intracellular fluid and cells alone. The results indicated that the supernatants co-cultured with the two *Bacillus* strains played a main role to the degradation of AFB_1_. The supernatants of *Bacillus* sp. H16v8 and *Bacillus* sp. HGD9229 also played a major role in the degradation of AFB_1_, yielding the degradation rates of 30.2% and 36.5%, respectively. The results showed that the degradation rate of co-culture supernatant was 87.7%, with 55.3% higher than that of *Bacillus* sp. H16v8 and *Bacillus* sp. HGD9229, respectively. The co-culture of two *Bacillus* strains enhanced the degradation rate of AFB_1_.

After 72 h of incubation, the AFB_1_ degradation from the extracellular extract of *Rhodococcus erythropolis* liquid culture reached to 66.8%. In addition, the active components in the culture supernatant have been inferred to be enzymatic [15]. After 72 h of incubation, the AFB_1_ degradation by the supernatant of *Bacillus licheniformis* CFR1 reached 93.57%, possibly because enzymes were involved in the degradation [27]. These findings also suggested that the degradation of AFB_1_ resulting from the extracellular proteins and perhaps the enzymes rather than the physical adsorption to the cells wall [28]. As shown in Figure 2, we found that compared to the cell lysate and cells, the AFB_1_ degradation was highest in the supernatant.

### 2.5. Effects of AFB_1_ Degradation by the Supernatant with Heat Treatment and Proteinase K

**Figure 3:**
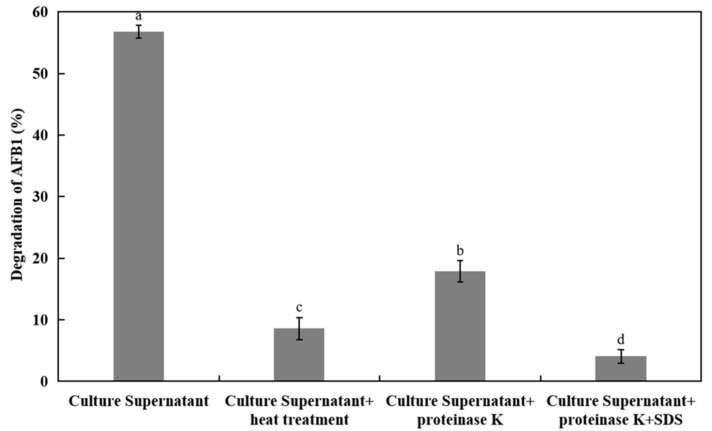
Degradation of AFB_1_ with the cell-free culture supernatant from mixed *Bacillus* cultures with or without heat, proteinase K treatment, and SDS. The samples were cultured at 37 °C for 12 h in the dark. The values were the average of three replicates and their standard errors. The mean values with the different letters were significantly different according to the Duncan’s multirange test (*p* < 0.05).

In Figure 3, after the incubation for 12 h, the culture supernatants could degrade AFB_1_ of about 56.7%. After culture supernatants were disposed by the heat treatment, the degradation rate of AFB_1_ was decreased by 85%. After culture supernatants were treated with proteinase K plus SDS, the AFB_1_ degradation decreased by 92.8% significantly. The results showed that treatments with heating and proteinase K with or without SDS obviously reduced with eradicated the degradation rate of supernatants. It demonstrated that the proteins/enzymes were employed in the degradation by mixed bacterial cultures.

On various occasions, microbial biodegradation of mycotoxins is primarily attributable to enzyme activity. After 72 h culture, with the culture supernatant of *Bacillus subtilis* JSW-1, the degradation rate of AFB_1_ was 62.8%, which was confirmed to be enzymatic by treatment with proteinase K treatments [29]. When proteinase K+SDS was used to treat the supernatant of *Pseudomonas* N17-1, the degradation rate was significantly reduced by 34.0% [30]. These findings also released that the degradation of AFB_1_ was because of the extracellular enzymes mixed *Bacillus* cultures. In order to further verify the detoxifying enzymes in culture supernatant, it is necessary to study the effects of temperature, pH, and metal ions on AFB_1_ degradation by supernatant.

### 2.6. Effects of AFB_1_ Degradation by the Supernatant with Different Metal Ions, pH, and Temperature

**Figure 4:**
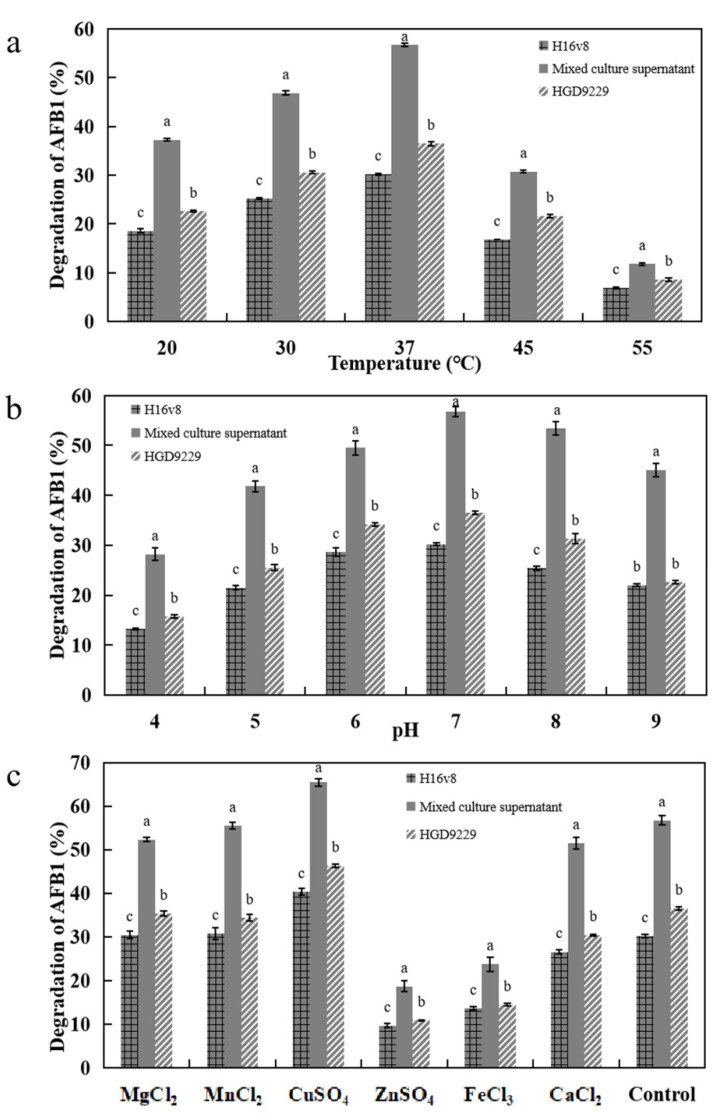
Effects of cultured temperature (**a**), pH (**b**), and metal ions (**c**) on the degradation of AFB_1_ by the culture supernatant of H16V8, HGD9229 and mixed cultures after 12 h. (**a**) Effect of temperature on the degradation of AFB_1_ by the culture supernatant of H16V8, HGD9229 and mixed cultures. (**b**). Effect of pH on the degradation of AFB_1_ by the culture supernatant of H16V8, HGD9229 and mixed cultures. (**c**) Effect of metal ions on the degradation of AFB_1_ by the culture supernatant of H16V8, HGD9229 and mixed cultures. The initial concentration of AFB_1_ was 100 μg/L and 12 h culture. The values were the average of three replicates and their standard errors. The mean values with the different letters were significantly different according to the Duncan’s multirange test (*p* < 0.05).

In Figure 4a, with the increase in temperature, the degradation rate gradually increased and reached its maximum at 37 °C. The supernatants from mixed and pure cultures were the same optimum degradation temperature. It was discussed that 37 °C should be more appropriate for both growth and enzymes production, similarly [31].

In Figure 4b, the highest degradation rate of AFB_1_ (56.73%) was achieved at pH 7.0, and the lowest degradation rate of AFB_1_ (28.21%) was achieved at pH 4. In agreement with previous literature descriptions, AFB_1_ degradation capacity was much higher in mixed culture conditions than in pure culture at different pH values [27]. The enzyme has an optimal pH range for maximum activity. In the enzymatic reaction, the correlation between AFB_1_ degradation and pH was typical [32].

In Figure 4c, it was found that metal ions have a significant effect on the degradation of AFB_1_. For example, Cu^2+^ stimulated the degradation of AFB_1_; Mn^2+^ and Mg^2+^ had no apparent influence. In addition, Fe^3+^ and Zn^2+^ had the inhibition effects yet. These results indicated that Cu^2+^ as an enzyme activator and membrane stabilizer may be beneficial to maintaining the structural integrity of proteins [33]. The form of affinity for AFB_1_ was reduced because of a conformational change of the enzyme by Zn^2+^ [6]. The influence of metal ions on the activity of mixed bacteria further enhanced the degradation of AFB_1_ by the enzyme [34].

### 2.7. Effect of Detoxification Time on AFB_1_ Degradation Rate

**Figure 5:**
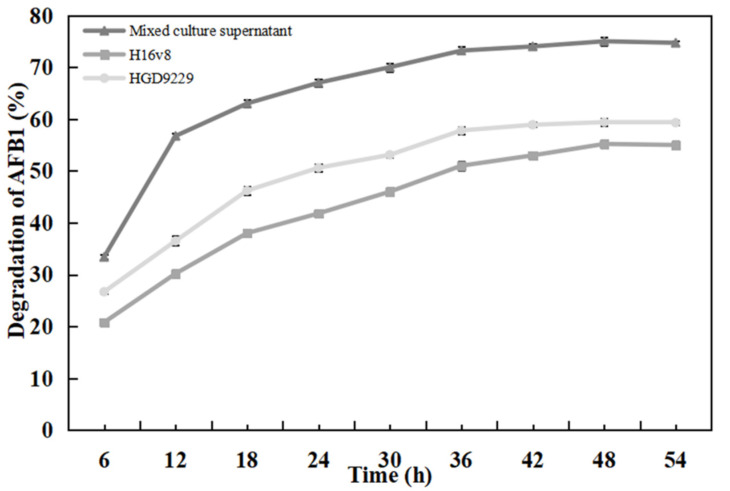
Time course of in vitro effect of detoxification time on degradation of AFB_1_ (initial concentration 100 μg/L) by culture supernatant of *Bacillus* sp. H16v8 and *Bacillus* sp. HGD9229 after 12 h incubation at 37 °C. The values were the average of three replicates and their standard errors. The mean values were significantly different according to the Duncan’s multirange test (*p* < 0.05).

As shown in Figure 5, the supernatants of *Bacillus* sp. H16v8, *Bacillus* sp. HGD9229, and their co-cultures were detoxified under different AFB_1_ degradation time conditions. The results indicated that the degradation rate increased with the degradation time. Additionally, the degradation rate presented a trend of rapid increase first and then gradually decreased. The degradation rate of AFB_1_ with the co-culture supernatant for 12 h was 56.7%. The degradation rate of AFB_1_ was 73.2%, when the detoxification time extended to 36 h. Consequently, the detoxification time of the co-culture supernatant of 12 h as the optimum treatment time could shorten much of the degradation time and has a practical application value. When the detoxification time was 12 h, the degradation rate of *Bacillus* sp. H16v8 and *Bacillus* sp. HGD9229 was 30.2% and 36.5%, respectively. Compared with the degradation rate with *Bacillus* sp. H16v8 and *Bacillus* sp. HGD9229, the degradation rate of co-culture supernatant was increased by 87.7% and 55.3%, respectively. In order to further expound the active components of the enzyme in the supernatant, it is necessary to determine the molecular weight (MW) range of the key detoxification enzymes.

### 2.8. Determination of the Molecular Weight Range for the Effective Enzymes

In Table 1, the supernatants with a MW of 10–30 kDa showed the higher degradation of AFB_1_ compared to the other MWs. The results showed that the MW range of the main detoxifying enzymes was 10–30 kDa. In Table 2, the supernatant with a MW of 10–30 kDa in the mixed cultures contained the higher concentrations of protein than that of the two *Bacillus* strains individually. It is determined that the co-culture of the two strains increased the protein contents in the 10–30 kDa. The co-culture of two bacteria increased the detoxifying enzymes production within 10–30 kDa to enhance the degradation ability of AFB_1_. It was found that in order to further improve the yield and enzymatic hydrolysis performance of cellulase, the mixed culture of the recombinant strains of *Trichoderma reesei* and *Aspergillus niger* was employed [35].

**Figure 6:**
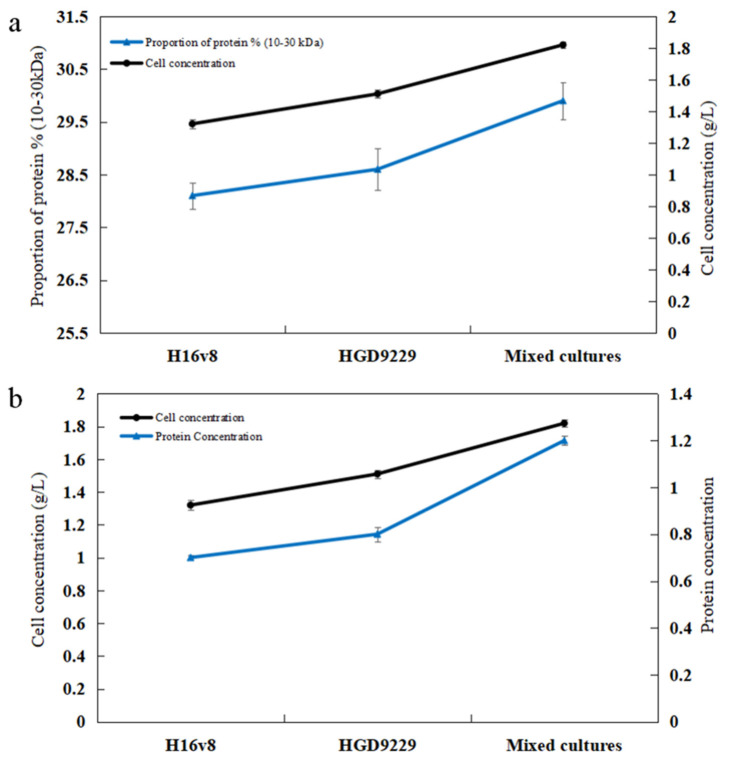
Relationship between protein content and cell concentration. (**a**) Relationship between cell concentration and proportion of protein (10–30 kDa). (**b**) Relationship between cell concentration and total protein concentration. Samples were incubated at 37 °C for 12 h. The values were the average of three replicates and their standard errors. The mean values were significantly different according to the Duncan’s multirange test (*p* < 0.05).

In Figure 6a, the proportion of protein (10–30 kDa) from mixed bacterial cultures was higher than that from pure culture. The increase in this proportion was positively correlated with cell concentration. The synergistic interactions of the two *Bacillus* species promoted cell growth, which was responsible for the increased proportion of detoxifying enzymes. Particularly, the synergistic effect of *Agaricus arvensis* and *Sistotrema brinkmannii* resulted in several times higher total enzyme activity than when cultured alone [36]. The increase in cellulase and xylanase activity was displayed by the affinity interaction between *Irpex lacteus* and *Schizophyllum Commune* [37]. The cellulase derived from *Trichoderma reesei* exhibited low β-glucosidase activity [38]. The simplest method was to add the β-glucosidase of *Aspergillus niger* to the cellulase of *Trichoderma reesei*. To further increase cellulase production, a mixed cultivation of recombinant *Trichoderma reesei* and *Aspergillus niger* was employed [35]. Moreover, it is suggested that the cell concentration from mixed cultures is related to the production of the enzymes [39].

The initial concentration of AFB_1_ was 100 μg/L. Samples were incubated at 37 °C for 48 h. The culture supernatant was concentrated by super filter with cut-off MW of 3 kDa (Millipore). In total,10 mL of the pure and mixed culture supernatant was concentrated to 1 mL and used for AFB_1_ degradation.

The concentration of proteins in the culture supernatant from the mixed bacterial cultures was higher than that of the culture supernatant from the two *Bacillus* sp. strains individually (Figure 6b). In Table 3, the maximum degradation rate of AFB_1_ (85.2%) was obtained in the supernatant of mixed bacterial cultures, which was more effective than those of the pure cultures individually at the same conditions. It was shown that the AFB_1_ degradation by the culture supernatant of *Bacillus* sp. H16v8, *Bacillus* sp. HGD9229 and mixed bacteria increased by 38.7%, 30.4%, and 50.3% after ultrafiltration. The AFB_1_ degradation was enhanced with the increase in protein concentrations (Table 3). It is in agreement with the literature that the degradation of aflatoxin with the culture supernatant by *P. aeruginosa* N17-1 enhanced by 46.3% after the ultrafiltration [30]. The cell-free extracts of the strains with higher protein concentrations obtained a higher AFB_1_ degradation rate [13]. It is found that the co-cultivation for two *Bacillus* strains is a useful way to degrade AFB_1_.

**Figure 7:**
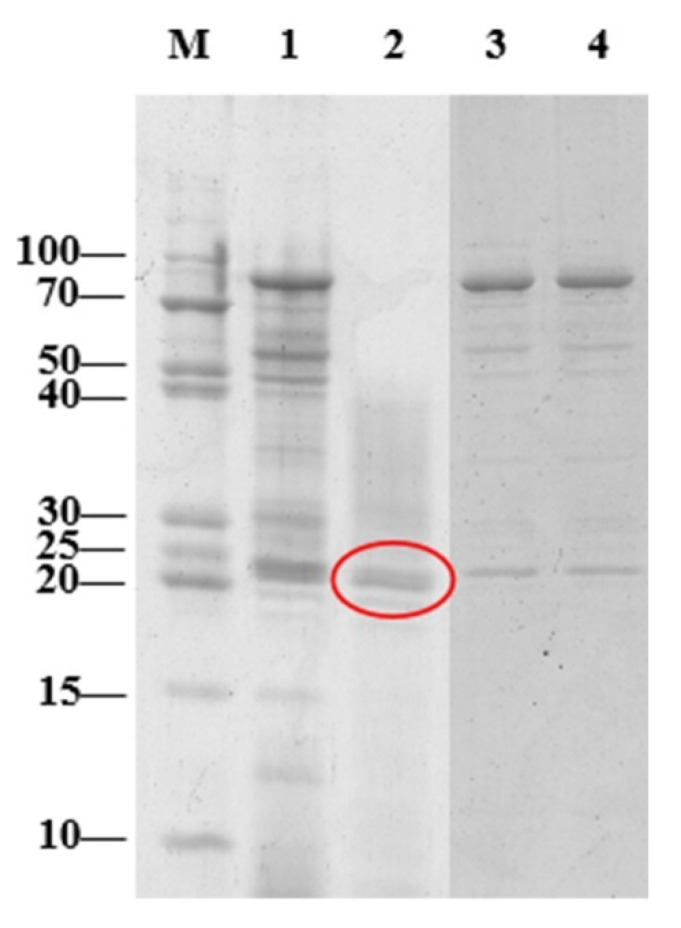
SDS-PAGE analysis of proteins in the mixed bacterial cultures and separate species. (**M**) protein marker, (**1**) The culture supernatant (10 mL) from mixed bacterial cultures was enriched by super filter with cut-off molecular weight (MW) of 10 kDa and concentrated to 1 mL. (**2**) The culture supernatant (10 mL) from mixed bacterial cultures was separated by super filter with cut-off MW of 30 kDa, then transferred the filtrate to a 10 kDa ultrafiltration tube and concentrated to 1 mL. (**3**) The culture supernatant (10 mL) from *Bacillus* sp. H16v8 was enriched by super filter with cut-off MW of 10 kDa. (**4**) Culture supernatant (10 mL) of *Bacillus* sp. HGD9229 was enriched by super filter with cut-off MW of 10 kDa (Millipore).

In Figure 7, the SDS-PAGE of 10–30 kDa protein sample was shown in line 2. It was further found that line 2 (20–25 kDa) had a strong AFB_1_ degradation ability, which was consistent with the literature that the results of SDS-polyacrylamide gel electrophoresis showed the MW (22 kDa) of the enzyme extracted from the supernatant of *Bacillus shackletonii* L7 [33]. On the same electrophoresis volume, with the higher of protein contents, the bands were darker. Under the same loading volume conditions, the bands depth ranged from 20 to 25 kDa, which has obviously increased. In addition, this MW enzyme demonstrated the most power of influence on the degradation efficiency. Other MW bands showed increased depth as well. The total value of electrophoresis bands saw the increased trend. The choice of nitrogen source was closely related to protein production [40]. Similarly, the choice of yeast extract as a nitrogen source was one of the possible reasons for the improvement of enzyme production with the mixed bacterial cultures. The cell concentration of pure and mixed bacterial cultures also had a positive correlation with total protein concentration. From Figure S2b, the co-cultivation for two *Bacillus* strains promoted the total growth. It was also found that *Bacillus* sp. H16v8 and *Bacillus* sp. HGD9229 mutually promoted the production of total protein from culture supernatant (Table 3). At the same time, our research determined that the co-culture of two *Bacillus* strains led to an increase in detoxifying enzymes production in 20–25 kDa. During the degradation of bamboo-shoot shell powder, the increase in activities of enzymes carboxymethyl cellulase and laccase by co-cultivation of *Pleurotus ostreatus* and *Aspergillus niger* could be observed [41]. Similarly, the activity laccase was enhanced by the co-culture of *T. versicolor* and *A. niger* [42]. Therefore, the enzyme mixture co-cultivated with fungi was may be more effective for industrial applications than the enzyme from the mono-cultivations [43]. In this study, it was found that the degradation reaction associated with enzymes of molar mass in the range 20–25 kDa. The co-culture of two *Bacillus* species in liquid medium increased the production of AFB_1_ detoxifying enzymes to enhance the ability of the AFB_1_ degradation.

### 2.9. Degradation Pathways of AFB_1_ by Enzymes from the Mixed Cultures

**Figure 8:**
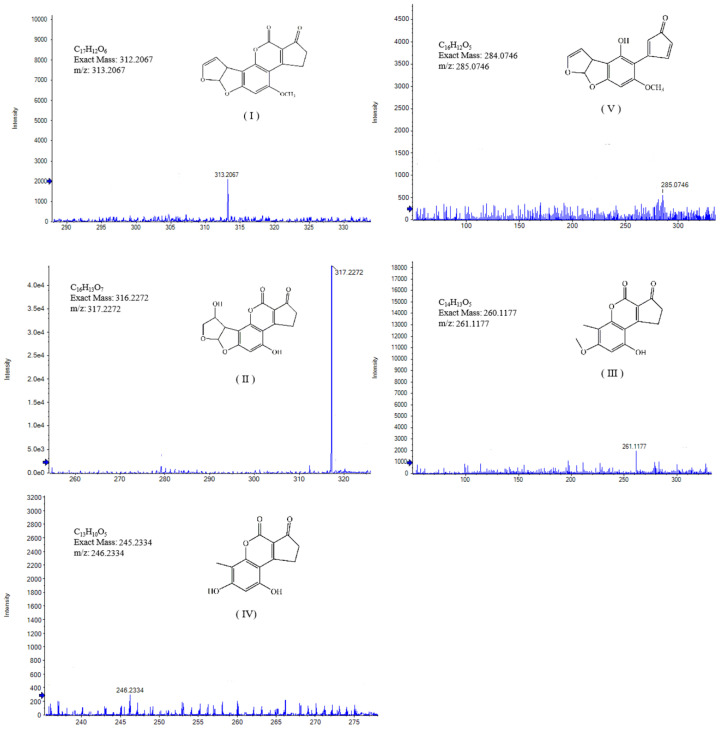
Mass spectrum of AFB_1_ and the four key products in the two different degradation pathways of mixed bacterial cultures. The ion peaks values of theses constituents were at *m/z* 313.2067 (C_17_H_12_O_6_, Ⅰ), 285.0746 (C_16_H_12_O_5_, Ⅴ), 317.2272 (C_16_H_13_O_7_, Ⅱ), 261.1177 (C_14_H_13_O_5_, Ⅲ), and 246.2334 (C_13_H_10_O_5_, Ⅳ). Structures of these products were only detected in degradant produced by the supernatant of mixed cultures.

These samples include degradation products and controls/blanks (cell solution without AFB_1_) and were analyzed by LC-Triple TOF MS to determine the uncertain composition in the degradation products. The detected composition in both degradation products and controls with the ion value of *m/z* 313.2067 was AFB_1_ (C_17_H_12_O_6_, Ⅰ). Figure 8 shows the mass spectrum of AFB_1_ and the four key products in the hypothetical degradation pathway of mixed bacterial cultures. The ion peak values of these components were at *m/z* 285.0746 (C_16_H_12_O_5_, Ⅴ), 317.2272 (C_16_H_13_O_7_, Ⅱ), 261.1177 (C_14_H_13_O_5_, Ⅲ), and 246.2334 (C_13_H_10_O_5_, Ⅳ), which were only detected in degradant produced by the supernatant of mixed cultures. Therefore, these results indicated that AFB_1_ was decomposed by biodegradation, and four products with structures that were significantly different from AFB_1_ could be obtained.

The co-cultivation of two *Bacillus* strains enhanced the production of detoxifying enzymes, which increased the diversity of AFB_1_ degradation products and degradation pathways. Among them, the component with *m/z* of 317.0653(Ⅱ) was detected in the product of degradation of AFB_1_ with radiolysis [44]. This result was probably caused by an additional reaction between a hydroxyl group on the double bond of the furan ring on the left and a methyl group on the side chain of the AFB_1_ benzene ring [44]. It was reported that the degradation rate of AFB_1_ was 95.21% by *Corymbia citriodora* aqueous extracts at the initial concentration of 100 μg/L. The degradation product also contained the constituent with *m/z* 285.0746(Ⅴ) by LC-MS/MS [45]. The results show that AFB_1_ could be effectively decreased by aqueous ozone with the constituent of *m/z* 261.1177(Ⅲ), respectively [46]. The constituents with *m/z* 246.2334(Ⅳ) identified in the degradation products of AFB_1_ with high-voltage atmospheric cold plasma (HVACP) [47].

**Figure 9:**
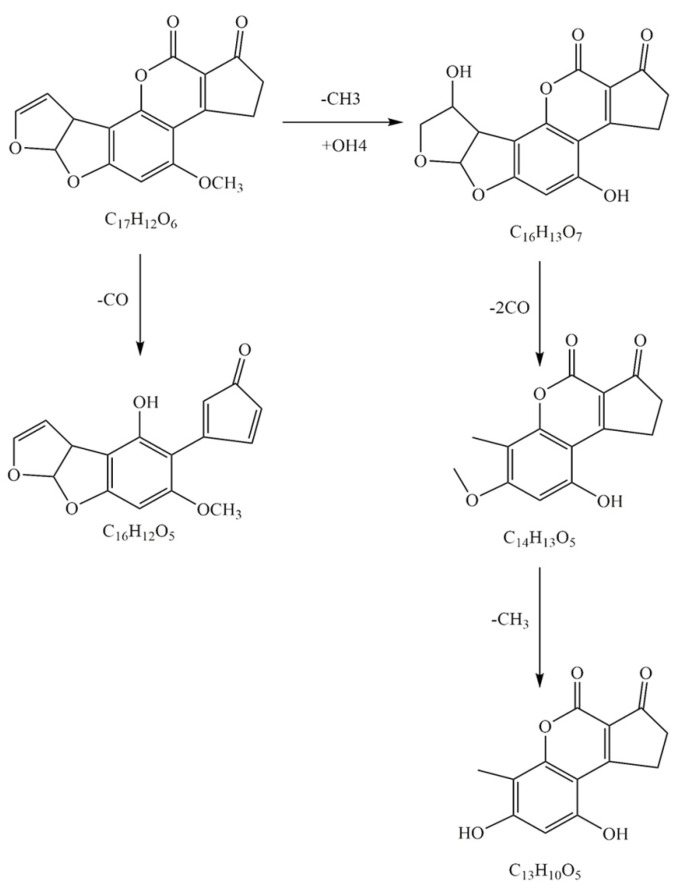
The hypothetical pathways of AFB_1_ degradation by mixed bacterial cultures. Two pathways of AFB_1_ degradation were inspired by the structural formula of the four degradation products and previous works.

The co-culture of two *Bacillus* strains promoted the production of enzymes and generated further degradation of AFB_1_, thus obtaining richer degradation mechanisms. As shown in Figure 9, the degradation pathway of AFB_1_ in mixed bacterial cultures could be inferred from the literature [44,46,47]. There are two possible biodegradation pathways for AFB_1_. First, the degradation product *m/z* 317.0653 (Ⅱ) was determined by the double bond located in the left side of the furan ring and caused an additional reaction of one hydroxyl groups. Second, the methoxy group on the side chain of the benzene ring is replaced by the hydroxyl group. Then, two carbon monoxide (CO×2) were lost in the furan ring after hydrolysis of AFB_1_, and the metabolite of *m/z* 261.1177 (C_14_H_13_O_5_, Ⅲ) was obtained. A methyl group was missing from the side chain of the benzene ring, and the metabolite with *m/z* of 246.2334(Ⅳ) was obtained. The other was a compound with *m/z* of 285.0746 (C_16_H_12_O_5_, Ⅴ), which had one less CO molecule than AFB_1_ and was involved in ester bond fracture. In our study, the disappearance of blue fluorescence after the degradation of AFB_1_ caused our attention. It was demonstrated that the treatment of AFB_1_ with enzymes altered the rupture of the lactone ring by reduction, with consequent changes in the fluorescence and mutagenicity of the molecule [15].

AFB_1_ contains a furan moiety and a lactone ring, and the key active site of AFB_1_ with carcinogenesis and toxicity was the double bond on the furan ring at its terminal. The reduced toxicity of the radioactive product *m/z* 317.0653 (II) was reported because of the addition reaction on the terminal furan ring double bond [44]. Compared with the AFB_1_, the toxicity of these degradation products (Ⅲ) 261.1177 *m/z* and *m/z* 246.2334 (Ⅳ) were markedly reduced, because the double bond in the terminal furan ring had been removed [7]. By observing the changes of AF molecular lactone ring [16], gas chromatography mass spectrometry (GC-MS) and Fourier-transform infrared spectroscopy (FT-IR) analysis revealed that AFB_1_ was converted into AFD_1_ after degradation. The results showed that the toxicity of the degraded compounds was much lower than that of AFB_1_. By the brine shrimp bioassay biological toxicity of AFB_1_ degradation product analysis, *m/z* 285.0746 degradation compounds (Ⅴ) of the brine shrimp larvae toxicity were far lower than the AFB_1_ [45]. All these results proved that the mixed bacterial culture could effectively degrade AFB_1_.

## 3. Conclusions

In summary, the two strains that could detoxify AFB_1_ were isolated and identified as *Bacillus velezensis* H16v8 and *Bacillus megaterium* HGD9229. At the temperature of 37 °C and the action time of 12 h, with the initial AFB_1_ concentration of 100 μg/L, the supernatant from mixed cultures of the two *Bacillus* showed the AFB_1_ degradation of 56.7%. The degradation efficiency of the supernatant from the mixed cultures increased by 87.7% and 55.3%, compared to *Bacillus* sp. H16v8 and *Bacillus* sp. HGD9229, individually. Additionally, the active proteins component has been determined as the MW of 20–25 kDa. The co-culture of two *Bacillus* strains promoted the growth of the cells and the production of extracellular proteins, thus increasing the production of detoxifying enzymes in the 20–25 kDa range. The degradation of AFB_1_ may involve two pathways: it may either be directly translated into V (C_16_H_12_O_5_) constituent or be translated to form IV (C_13_H_10_O_5_) through II (C_16_H_13_O_7_) and III (C_14_H_13_O_5_). It is speculated that the degradation of AFB_1_ is related to the destruction of lactone ring and furan ring. The four products were all less or not toxic according to the literature. The method of mixed culture provided a novel way to enhance AFB_1_ detoxification, which was considered a practical choice and the environmentally friendly process for AFB_1_ degradation in food and feed industry.

## 4. Materials and Methods

### 4.1. Chemicals and Medium

AFB_1_ standard was obtained from Pribolab (Qingdao, China). Coumarin medium (CM) was prepared according to the literature [31].

### 4.2. Isolations of AFB_1_-Degrading Bacteria

#### 4.2.1. Bacteria Isolations

Aflatoxins are a set of coumarin derivatives; thus, microorganisms that can use coumarin as a carbon source can also use or metabolize aflatoxin [29]. A dozen soil samples and several fecal samples of the cows were collected from farmland near Henan University of Technology (Zhengzhou, Henan, China). The selective culture method with the primary and second screening were performed by the method of the literature [29]. The selected strains were temporarily preserved in 20% glycerol solution at −20 °C. The typical characteristics of the selected stains were observed under a biological microscope (BK 5000, China).

#### 4.2.2. Molecular Identification of the Isolates

The molecular identification of the selected strains was performed to analyze the gene sequence and nucleotide sequence of the target strain [29] and to construct the phylogenetic tree.

### 4.3. Tests of AFB_1_ Degradation

The isolated strain was cultured in the NB at 37 °C for 48 h on the shaker of 180 r/min. AFB_1_ standard solution was diluted with methanol (Beijing Chemical Inc., Beijing, China) to a stock solution of 10 μg/mL. After being mixed with the culture medium of mixed bacteria, the initial concentration of AFB_1_ was 100 μg/L. Then, the mixed solution was performed at 37 °C in static and dark for 12 h. The AFB_1_ standard solution added to the sterile NB without stains in the initial AFB_1_ concentration of 100 μg/L at the same condition served as the control. The contrastive objects were supplemented by the detoxification with the *Bacillus* sp. H16v8 culture and *Bacillus* sp. HGD9229 culture. At the end of incubation, samples were collected using the method described in literature [27] and analyzed by thin-layer chromatography (TLC) [48] and high-performance liquid chromatography (HPLC) analysis [49].

In total, 10 μL chloroform extract were spotted on TLC plate (Qingdao Haiyang chemical Co., LTD, China) and developed in chloroform: acetone: water (88:12:1, *v/v/v*). The chromatogram was observed under 365 nm UV light. The AFB_1_ was analyzed using the Agilent 1260 HPLC. HPLC was performed by an Agilent C_18_ column. The C_18_ column was 250 × 4.6 mm with a particle size of 5 µm. The mobile phase was methanol: water (1:1, *v/v*) at a flow rate of 1 mL/min. AFB_1_ was derived from a photochemical reactor (Hua Mei Chen Equipment Co., LTD., Suzhou, China) and measured by a fluorescence detector [27]. The standard curve of AFB_1_ by HPLC was shown in Figure S4. The standard curve equation was y = 1.0336x−2.794, R^2^ = 0.9993. The AFB_1_ degradation rate calculation formula was as follows:

$$(1-\frac{{\mathrm{AFB}}_{1}\;\mathrm{peak}\;\mathrm{area}\;\mathrm{in}\;\mathrm{treatment}}{{\mathrm{AFB}}_{1}\;\mathrm{peak}\;\mathrm{area}\;\mathrm{in}\;\mathrm{control}})\;\times \;100\%$$

### 4.4. AFB_1_ Degradation by Mixed Bacterial Cultures

The isolates were cultured in the NB for 24 h. Then, two different bacteria were chosen to construct the novel mixed bacterial cultures. Additionally, 2.5 mL culture broth for each pure bacterial was added into 50 mL NB. The mixed bacterial cultures were cultured at 37 °C, 180 r/min and taken out at 6, 12, 18, 24, 30, 36, 42, 48, 54, and 60 h. Then, the mixed bacterial cultures were collected and tested for AFB_1_ degradation. The tests of AFB_1_ degradation were performed as referred to at 4.3. The cell dry weights of pure and mixed bacterial cultures were measured in 6, 12, 18, 24, 30, and 36 h. The growth curves of mixed cultures were determined by OD600. The biomass was determined by the previous report [50].

### 4.5. AFB_1_ Degradation by Cells, Culture Supernatant, and Cell Lysate

*Bacillus* sp. H16v8 and *Bacillus* sp. HGD9229 were cultured in the NB for 24 h. Then, both were chosen to construct the novel mixed bacterial cultures. Additionally, 2.5 mL culture broth for each pure bacterial was added into 50 mL NB. After cultured at 37 °C for 12 h on the shaker of 180 r/min, the cells were obtained by centrifuge with the condition of 8000× *g* for 10 min at 4 °C. The culture supernatant was gathered for the degradation of AFB_1_ [30].

The cells were washed twice and then suspended with the phosphate buffer (50 mM) at pH 7.0. The ultrasonic cell disintegrator was performed to extract intracellular extracts for the further experiments [27,33].

### 4.6. The Effects of Heat Treatment, Proteinase K, Metal Ions, Temperature, and pH on AFB_1_ Degradation by the Supernatant

The supernatant was immersed in a boiling water bath for 10 min. The effect of protease treatment was determined after the culture by the previous literature [31].

To evaluate the influence of different metal ions on the reaction mixture, Mg^2+^, Mn^2+^, Cu^2+^, Zn^2+^, Fe^3+^, and Ca^2+^ (in the form of MgCl_2_, MnCl_2_, CuSO_4_, ZnSO_4_, FeCl_3_ and CaCl_2_) were, respectively, added to the reaction mixture with the final ion concentration of 10 mM. The NB substituted the supernatant in the control. The effects of temperature (20, 30, 37, 45, and 55 °C) and pH (4.0, 5.0, 6.0, 7.0, 8.0, and 9.0) on the degradation of AFB_1_ by supernatant.

### 4.7. Active Fraction of SDS Polyacrylamide Electrophoresis

The culture supernatants were concentrated by ultrafilter, and the cut-off MWs were 3, 10, and 30 kDa (Millipore), respectively. SDS-PAGE analysis of proteins was performed on the culture supernatant according to the method in literature [51]. The above ultrafiltration protein concentrate sample was mixed with 5 × SDS protein electrophoresis loading buffer at 4:1, heated at 100 °C for 5 min, and centrifuged at 7690× *g* for 10 min. The pageruler unstained protein ladder 26,614 (10–200 kDa, Thermo Scientific, Lithuania). The system (Biorad, Segrate, Italy) was filled with the buffers composed of 25 mM Tris and 0.19 M glycine and used for 30 min at 80 V and 80 min at 120 V. Then, the gel was stained with 0.25% (*w/v*) Coomassie Brilliant Blue R-250 and destained in 7% (*v/v*) acetic acid. Protein concentration of the culture supernatant was determined by the Bradford method [52].

### 4.8. Structures of Degradation Products of AFB_1_ treated by Detoxifying Enzymes Treatment

The supernatant of 950 μL mixed culture bacteria was collected. AFB_1_ standard solution with a final concentration of 500 ppb was added into the supernatant according to the literature [27]. The sample was incubated at 30 °C for 24 h in the dark. Groups without AFB_1_ were added as blank. The samples were pretreated and analyzed by LC-Triple TOF MS according to the method in the literature [19]. The degradation products of AFB_1_ were detected by SHIMADZU Nexera X2 HPLC (SHIMADZU, Japan). Chromatography was performed with a 150 mm × 2.1 mm and 2.6 μm C_18_ 100 Å Phenomenex Kinetex HPLC column. The injection method was described by the literature [19]. The mobile phases were water containing 0.1% formic acid and acetonitrile containing 0.1% formic acid.

MS was performed with an AB SCIEX Triple TOF 6600 mass spectrometer. The optimized conditions were as follows: data were acquired using an ion spray voltage of 5.5 kV in positive ionization mode, nebulizer gas (gas 1), 50 psi; auxiliary gas (gas 2), 50 psi; ion-source temperature, 500 °C; curtain gas, 30 psi. The mass range of the survey scan was 50–1000 *m/z*, and the accumulation time was 500 ms. 

### 4.9. Statistical Analysis

SAS (SAS Institute, Cary, NC, USA) general linear model program was adopted, and ANOVA was used to design completely random single factor. The Duncan’s multirange test could be applied to determine whether the discrepancies among the several groups were at a statistically significant level of 0.05 and was used for mean comparison [53]. The values were the average of three replicates and their standard errors. The mean values with the different letters were significantly different according to the Duncan’s multirange test (*p* < 0.05).

## Figures and Tables

**Table 1:** Degradation rate of AFB_1_ in different parts of the supernatant at same concentration.

Strain	Degradation (%)/0.1 mg <10 kDa ^1^	Degradation (%)/0.1 mg 10–30 kDa ^2^	Degradation (%)/0.1 mg >30 kDa ^3^
H16v8	2.9 ± 0.31	20.8 ± 0.61	2.6 ± 0.21
HGD9229	3.2 ± 0.22	21.3 ± 0.46	2.8 ± 0.32
Mixed cultures	4.1 ± 0.15	24.5 ± 0.52	2.7 ± 0.25

^1^ In total, 10 mL of the filtrate was obtained with a 10 kDa ultrafiltration tube. Then, the filtrate was transferred to the ultrafiltration tube of 3 kDa and concentrated to 1 mL. ^2^ In total, 10 mL of the filtrate was obtained with the ultrafiltration tube of 30 kDa. Then, the filtrate was transferred to the ultrafiltration tube of 10 kDa and concentrated to 1 mL. **^3^** In total, 10 mL supernatant was concentrated to 1 mL with the ultrafiltration tube of 30 kDa. All the samples were diluted with sterile water to 0.1 mg/mL for AFB_1_ degradation. The values were the average of three replicates and their standard errors. The mean values were significantly different according to the Duncan’s multirange test (*p* < 0.05).

**Table 2:** Protein concentration of different ranges in the supernatant.

Strain	<10 kDa (mg/mL) ^1^	10–30 kDa (mg/mL) ^2^	>30 kDa (mg/mL) ^3^
H16v8	0.13 ± 0.03	0.16 ± 0.01	0.28 ± 0.02
HGD9229	0.15 ± 0.04	0.20 ± 0.02	0.35 ± 0.02
Mixed cultures	0.19 ± 0.02	0.29 ± 0.02	0.49 ± 0.03

^1^ In total, 10 mL of the filtrate was obtained with the ultrafiltration tube of 10 kDa, and then the filtrate was transferred to the ultrafiltration tube of 3 kDa and concentrated to 1 mL for protein concentration test. **^2^** In total, 10 mL of the filtrate was obtained with the ultrafiltration tube of 30 kDa, and then the filtrate was transferred to the ultrafiltration tube of 10 kDa and concentrated to 1 mL for protein concentration test. ^3^ In total, 10 mL supernatant was concentrated to 1 mL with the ultrafiltration tube of 30 kDa and used for protein concentration test. The values were the average of three replicates and their standard errors. The mean values were significantly different according to the Duncan’s multirange test (*p* < 0.05).

**Table 3:** Protein concentrations of supernatant from the pure and mixed bacterial cultures.

Strain	Protein Concentration (mg/mL)	Degradation (%)
*Bacillus* sp. H16v8	0.7 ± 0.03	58.4% ± 0.87
*Bacillus* sp. HGD9229	0.8 ± 0.04	60.1% ± 0.83
Mixed bacteria	1.2 ± 0.02	85.2% ± 1.04

The values were the average of three replicates and their standard errors. The mean values were significantly different according to the Duncan’s multirange test (*p* < 0.05).

## Data Availability

The data presented in this study are available within this article and its Supplementary Materials.

## Supplementary Materials

The following are available online at https://www.mdpi.com/article/10.3390/toxins13070435/s1, Figure S1. The two bacterial phylogenetic trees based on 16S rRNA gene sequence, Figure S2. Co-culture and cell dry weight test of two kinds of bacteria, Figure S3. Degradation of AFB_1_ by the cultures of *Bacillus* sp. H16v8, *Bacillus* sp. HGD9229 and mixed *Bacillus* in vitro from the initial AFB_1_ concentration of 100 μg/L, Figure S4. The standard curve of AFB_1_ by HPLC.
