# Supplementary material for: Co-Cultivation of Two Bacillus Strains for Improved Cell Growth and Enzyme Production to Enhance the Degradation of Aflatoxin B1
Source: Toxins (Basel). 2021 Jun 22;13(7):435. doi: 10.3390/toxins13070435 (PMC8309871; doi:10.3390/toxins13070435)
Supplement: Supplementary file 1 [file toxins-13-00435-s001.zip › toxins-1209139-supplementary.pdf]

Supplementary Materials: Co-Cultivation of Two *Bacillus* Strains for Improved Cell Growth and Enzyme Production to Enhance the Degradation of Aflatoxin B_1

Le Wang, Wei Huang, Yu Sha, Haicheng Yin, Ying Liang, Xin Wang, Yan Shen, Xingquan Wu, Dapeng Wu and Jinshui Wang

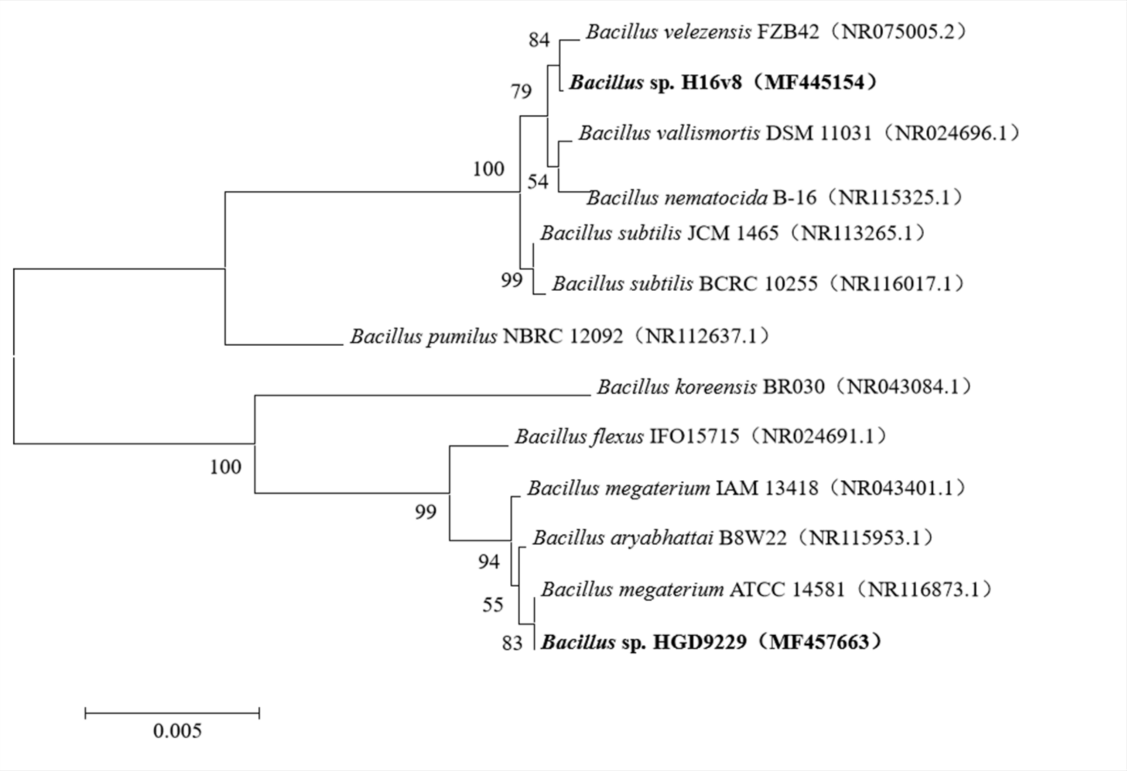

Figure S1. The two bacterial phylogenetic trees based on 16S rRNA gene sequence. The Neighbor-Joining (NJ) method was used to infer the evolutionary history. The number on the branch point refers to the self-service value based on 1000 resampling.

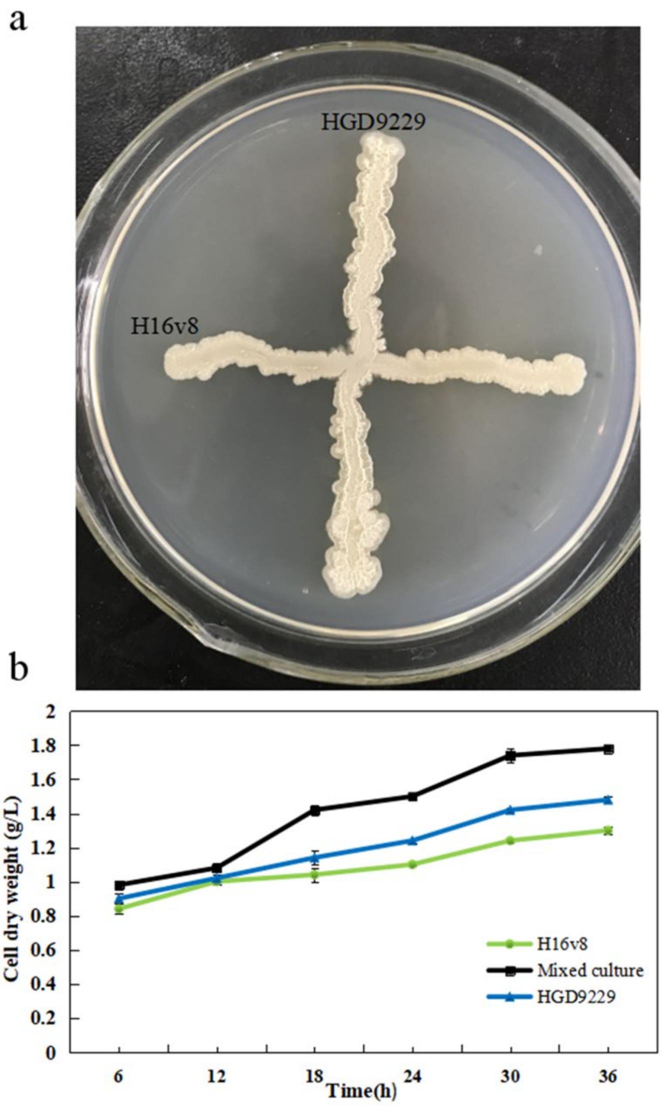

Figure S2. Co-culture and cell dry weight test of two kinds of bacteria. (**a**) *Bacillus* sp. H16v8 and *Bacillus* sp. HGD9229 were cross-inoculated on the same plate and cultivated at 37 °C for 24 h. Two kinds of bacteria cultured in NB medium. (**b**)**.** The cell dry weight at different fermentation times and the cultures performed at 37 °C.

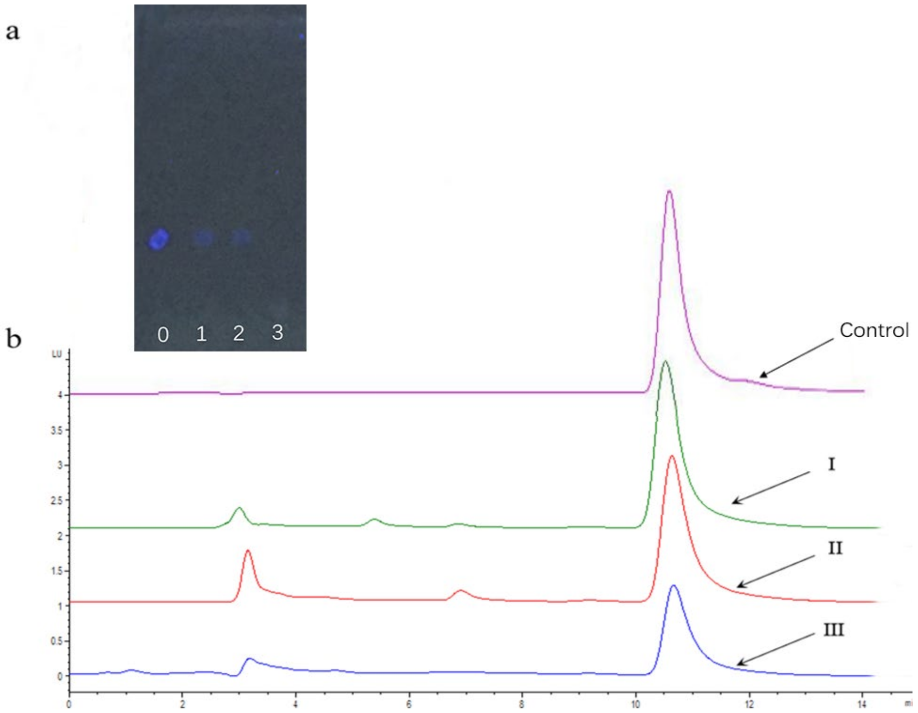

Figure S3. Degradation of AFB_1 by the cultures of *Bacillus* sp. H16v8, *Bacillus* sp. HGD9229 and mixed *Bacillus* in vitro from the initial AFB_1 concentration of 100 μg/L. (**a**) TLC plate showing degradation of AFB_1. Lane 0: The AFB_1 standard solution added to the sterile NB without stains after 12 h of incubation as the Control; Lane 1: the AFB_1 standard solution treated with *Bacillus* sp. H16v8 (the initial concentration of AFB1 was 100 μg/L) after 12 h of incubation; Lane 2: the AFB_1 standard solution treated with *Bacillus* sp. HGD9229 after 12 h of incubation; Lane 3: the AFB_1 standard solution treated with the mixed cultures of *Bacillus* sp. H16v8 and *Bacillus* sp. HGD9229 after 12 h of incubation. (**b**) HPLC chromatogram: Control: the AFB_1 standard solution added to the sterile NB without stains after 12 h of incubation; I: the AFB_1 standard solution treated with *Bacillus* sp. H16v8 after 12 h of incubation; II: the AFB_1 standard solution treated with *Bacillus* sp. HGD9229 after 12 h of incubation; III: the AFB_1 standard solution treated with the mixed cultures of *Bacillus* sp. H16v8 and *Bacillus* sp. HGD9229 after 12 h of incubation.

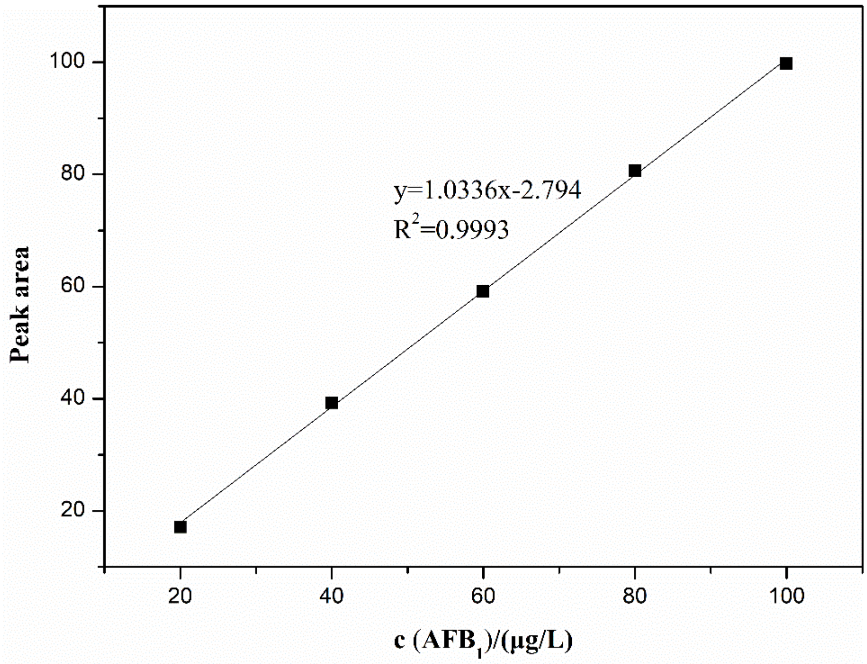

Figure S4. The standard curve of AFB_1 by HPLC. The peak area varied with the concentration of AFB_1. The concentration of AFB_1 was analyzed using the Agilent 1260 HPLC with the Agilent C_{18} column. AFB_1 was derived by a photochemical reactor and measured by a fluorescence detector.
